# Supplementary material for: Canu: scalable and accurate long-read assembly via adaptive k-mer weighting and repeat separation
Source: Genome Res. 2017 May;27(5):722–36. doi: 10.1101/gr.215087.116 (PMC5411767; doi:10.1101/gr.215087.116)
Supplement: Supplemental Material [file supp_27_5_722__index.html]

Canu: scalable and accurate long-read assembly via adaptive k-mer weighting and repeat separation — Canu: scalable and accurate long-read assembly via adaptive k-mer weighting and repeat separation — Supplemental Material 

# Canu: scalable and accurate long-read assembly via adaptive *k*-mer weighting and repeat separation

## Supplemental Material

- Supplemental\_Material.pdf
- Supplemental\_Code.tar.gz
