# Supplementary material for: Canu: scalable and accurate long-read assembly via adaptive k-mer weighting and repeat separation
Source: Genome Res. 2017 May;27(5):722–36. doi: 10.1101/gr.215087.116 (PMC5411767; doi:10.1101/gr.215087.116)
Supplement: Supplemental Material [file supp_gr.215087.116_Supplemental_Material.pdf]

Supplemental Material for "Canu: scalable and accurate long-read assembly via adaptive *k*-mer weighting and repeat separation"

Raw assemblies and polished results at http://gembox.cbcb.umd.edu/shared/canu/index.html

Supplemental Note 1: Kmer Filtering 3
Table S1: Overlap sensitivity with and without filtering....................3
Supplemental Note 2: Sequencing Data 4
Supplemental Note 3: Repeat separation simulation 5
Table S2: Assembly results for the two-copy repeat example....................6
Supplemental Note 4: Low Coverage Assembly 6
Table S3: Continuity and QV statistics *for A. thaliana* hybrid vs. hierarchical assemblies 7
Figure S1: Canu 20X+Quiver, Canu 20X+Pilon, and SPAdes 100X+20X and SPAdes 100X+150X *A. thaliana* assemblies 7
Supplemental Note 5: Assemblers 7
Falcon....................7
SPAdes....................7
Miniasm 8
Canu 8
Supplemental Note 6: Polishing 8
Supplemental Note 7: Validation 9
Supplemental Note 8: Assembler Runtimes....................9
Table S4: Canu assembly time (CPU hours)....................10
Table S5: Falcon assembly time (CPU hours) 10
Table S6. Miniasm assembly time (CPU hours)....................10
Table S7: SPAdes assembly time (CPU hours) 11
Supplemental Note 9: PacBio Assembly Statistics with Quiver 11
Table S8: Quiver Statistics for Canu 11
Table S9: Quiver Statistics For Falcon 11
Table S10: Quiver Statistics For Miniasm 11
Table S11: Quiver Assembly Statistics For SPAdes 12
Figure S2: Canu, Falcon, Miniasm, and SPAdes *E. coli* K12 polished assembles 12
Figure S3: Canu, Falcon, and Miniasm *D. melanogaster* assemblies....................12
Figure S4: Canu, Falcon, and Miniasm *A. thaliana* assemblies 12
Figure S5: Canu, Falcon, and Miniasm *C. elegans* assemblies....................13
Figure S6: Canu and Falcon assemblies of *H. sapiens* CHM1 13
Figure S7: Read length distributions for CHM1 P5-C3 (left) and HX1 (right) 14
Figure S8: Canu and published assemblies of *H. sapiens* HX1 14
Supplemental Note 10: Oxford Nanopore Assemblies with Nanopolish 15
Table S12: Nanopolish statistics for Canu....................15
Table S13: Nanopolish statistics for Falcon 15
Table S14: Nanopolish statistics for Miniasm....................15
Figure S9: Canu, Falcon, and Miniasm assemblies of *E. coli* K12 MAP005. 16
Figure S10: Canu, Falcon, and Miniasm assemblies of *E. coli* K12 MAP006-1. 16
Figure S11: Canu, Falcon, and Miniasm assemblies of *E. coli* K12 MAP006-2. 16

Figure S12: Canu, Falcon, and Miniasm assemblies of *E. coli* K12 MAP006-PCR-1. 17
Figure S13: Canu, Falcon, and Miniasm assemblies of *E. coli* K12 MAP006-PCR-2. 17
Figure S14: Canu, Falcon, and Miniasm assemblies of *B. anthracis*. 17
Figure S15: Canu, Falcon, and Miniasm assemblies of *Y. pestis*. 18
Supplemental Note 11: Oxford Nanopore assemblies with Pilon 18
Table S15: Pilon statistics for Miniasm after 300 rounds of polishing 18
Figure S16: Minimap remaining errors in *E. coli* K12 MAP006-1 19
Table S16: Pilon statistics for Canu 19
Table S17: Pilon statistics for Falcon 19
Table S18: Pilon statistics for Miniasm 19
Table S19: Pilon statistics for Spades 20
Figure S17: Canu, Falcon, Miniasm, and SPAdes assemblies of *E. coli* K12 MAP005 20
Figure S18: Canu, Falcon, Miniasm, and SPAdes assemblies of *E. coli* K12 MAP006-1 20
Figure S19: Canu, Falcon, Miniasm, and SPAdes assemblies of *E. coli* K12 MAP006-2 20
Figure S20: Canu, Falcon, Miniasm, and SPAdes assemblies of *E. coli* K12 MAP006-PCR-1 21
Figure S21: Canu, Falcon, Miniasm, and SPAdes assemblies of *E. coli* K12 MAP006-PCR-2 21
Figure S22: Canu, Falcon, Miniasm, and SPAdes assemblies of *B. anthracis.* 21
Figure S23: Canu, Falcon, Miniasm, and SPAdes assemblies of *Y. pestis.* 21
Supplemental Note 12: 1D Assembly 22
Figure S24: Canu 1D, Canu+three rounds of Nanopolish, Canu 1D+three rounds of Pilon, and Miniasm assemblies for *E. coli* K12 MAP006-1 1D. 22
Supplemental Note 13: *S. cerevisae* Nanopore assembly 22
Figure S25: Canu 1D, and Canu 1D+three rounds of Pilon, and Canu PacBio 115X assemblies. 23
Figure S26: Parallel overlap store construction 24
Figure S27: Suspicious read detection 24
References 25

Supplemental Note 1: Kmer Filtering

We used the *B. anthracis* Oxford Nanopore MinION dataset (Supplemental Note 2). All sequences were mapped to the reference *B. anthracis* str. 'Ames Ancestor' (GCF_000008445.1) with blasr (Chaisson and Tesler 2012):

```
blasr ba_filtered.fasta ref.fna -sa ref.sa -bestn 10  -maxAnchorsPerPosition 100 -
advanceExactMatches 10 -affineAlign -affineOpen 100 -affineExtend 0 -insertion 5 -deletion 5 -
extend -maxExtendDropoff 20 -nproc 8 -m 4 -out merged.blasr.m4
```

No significant mappings to pXO2 were found. k-mers predominantly coming from pXO1 were identified to have copy-number >40. Thus, all k-mers with >=40 occurrences were flagged as repeat and passed in a filter file to MHAP. We ran a sweep of suppression parameters including no suppression and full suppression and evaluated sensitivity on both pXO1 and the chromosome.

Table S1: Overlap sensitivity with and without filtering

Program	Filtering	Sensitivity on Chromosome	PPV on Chromosome	Sensitivity on pXO1	PPV on pXO1
MHAP	None	90.32%	99.52%	89.69%	99.55%
	0.1	90.30%	99.59%	87.63%	99.60%
	0.2	90.30%	99.62%	87.82%	99.66%
	0.3	90.30%	99.64%	87.86%	99.69%
	0.4	90.30%	99.61%	88.00%	99.65%
	0.5	90.30%	99.63%	88.15%	99.64%
	0.6	90.30%	99.56%	88.23%	99.65%
	0.7	90.30%	99.63%	88.53%	99.65%
	0.8	90.30%	99.57%	88.77%	99.60%
	0.9	90.30%	99.53%	89.05%	99.58%
	Full	89.68%	99.83%	26.34%	99.82%
MHAP sensitive	0.9	96.37%	92.96%	95.65%	93.02%
Daligner	Default	75.74%	100.00%	59.68%	100.00%
	-t1000 -M0	75.96%	99.98%	76.04%	99.98%
Minimap	Default	93.54%	89.22%	16.99%	89.23%
	-f 0.00000001	95.22%	84.63%	94.49%	84.64%

Overlaps were evaluated as in Berlin et al. (Berlin et al. 2015) using the commands:

```
java -cp mhap-2.1test.jar edu.umd.marbl.mhap.main.EstimateROC chromosome.m4 raw_reads.ovls
ba_filtered.fasta 2000 10000 true false 0.7 0.2 true
java -cp mhap-2.1test.jar edu.umd.marbl.mhap.main.EstimateROC pXO1.m4 raw_reads.ovls ba_filtered.fasta
2000 10000 true false 0.7 0.2 true
```

Minimap was run with the commands:

```
minimap -k 15 -Sw5 -L100 -m0 -t8 -I6G ba_filtered.fasta ba_filtered.fasta > minimap.paf
paf2mhap.pl ba_filtered.fasta minimap.paf > minimap.ovls

minimap -f 0.00000001 -k 15 -Sw5 -L100 -m0 -t8 -I6G  ba_filtered.fasta ba_filtered.fasta >
minimap.sens.paf
paf2mhap.pl ba_filtered.fasta minimap.sens.paf > minimap.sens.ovls
```

Daligner was run with the commands:

```
daligner -v -t16 -H1000 -e0.7 -s1000 raw_reads raw_reads
LAsort -v raw_reads.raw_reads.C0 raw_reads.raw_reads.N0 raw_reads.raw_reads.C1 raw_reads.raw_reads.N1
raw_reads.raw_reads.C2 raw_reads.raw_reads.N2 raw_reads.raw_reads.C3 raw_reads.raw_reads.N3 && LAmerge -v
```

```
raw_reads.1 raw_reads.raw_reads.C0.S raw_reads.raw_reads.N0.S raw_reads.raw_reads.C1.S
raw_reads.raw_reads.N1.S raw_reads.raw_reads.C2.S raw_reads.raw_reads.N2.S raw_reads.raw_reads.C3.S
raw_reads.raw_reads.N3.S && rm raw_reads.raw_reads.C0.S.las raw_reads.raw_reads.N0.S.las
raw_reads.raw_reads.C1.S.las raw_reads.raw_reads.N1.S.las raw_reads.raw_reads.C2.S.las
raw_reads.raw_reads.N2.S.las raw_reads.raw_reads.C3.S.las raw_reads.raw_reads.N3.S.las
LAshow raw_reads raw_reads.1.las > default.ovls

daligner -v -t1000 -H1000 -e0.7 -s1000 raw_reads raw_reads
LAsort -v raw_reads.raw_reads.C0 raw_reads.raw_reads.N0 raw_reads.raw_reads.C1 raw_reads.raw_reads.N1
raw_reads.raw_reads.C2 raw_reads.raw_reads.N2 raw_reads.raw_reads.C3 raw_reads.raw_reads.N3 && LAmerge -v
raw_reads.1 raw_reads.raw_reads.C0.S raw_reads.raw_reads.N0.S raw_reads.raw_reads.C1.S
raw_reads.raw_reads.N1.S raw_reads.raw_reads.C2.S raw_reads.raw_reads.N2.S raw_reads.raw_reads.C3.S
raw_reads.raw_reads.N3.S && rm raw_reads.raw_reads.C0.S.las raw_reads.raw_reads.N0.S.las
raw_reads.raw_reads.C1.S.las raw_reads.raw_reads.N1.S.las raw_reads.raw_reads.C2.S.las
raw_reads.raw_reads.N2.S.las raw_reads.raw_reads.C3.S.las raw_reads.raw_reads.N3.S.las
LAshow raw_reads raw_reads.1.las > sens.ovls
```

Truth overlaps were obtained from a bwa mem (Li 2013) mapping to the reference genome:

```
bwa mem -x ont2d b_anthracis.fna ba_filtered.fasta -t 16 samtools view -b -S - > mapping.bam
convertSam mapping.bam b_anthracis.fna > mapping.m4
cat mapping.m4 |grep NC_007530.2 > chromosome.m4
cat mapping.m4 |grep NC_007322.2 > pXO1.m4
```

Output was converted to BLASR's m4 format. Minimap overlaps were converted to MHAP format using the provided paf2mhap.pl script. Overlaps not found based on the reference mapping were confirmed using full Smith-Waterman alignment. If no alignment of at least 65% identity could be found, the overlap was considered a false-positive. While MHAP without filtering does not suffer the same PPV penalty as Minimap (due to its second-stage filter (Berlin et al. 2015) which checks the estimated mutation rate), performance suffers as the average number of sequences hit by a k-mer lookup increases from an average of 24.42 with filtering to 25.72 and the % of sequences which make it past the second-stage filter drops from an average of 78.29 to 77.06%. DALIGNER runtime increases 2-fold (from 1.28 CPU h to 2.58) and memory use increases 1.6-fold (from 38 GB to 61 GB).

Supplemental Note 2: Sequencing Data

Illumina MiSeq data for *E. coli* K12 was downloaded from the Illumina Scientific Data website (http://www.illumina.com/systems/miseq/scientific_data.html) and used for all analysis requiring Illumina data (Pilon polishing/SPAdes assembly). Illumina MiSeq *S. cereviase* W303 data was downloaded from Goodwin et al. (Goodwin et al. 2015) (http://schatzlab.cshl.edu/data/nanocorr/). Illumina MiSeq *A. thaliana* Ler-0 data was downloaded from Lee et al. (Lee et al. 2014) (http://schatzlab.cshl.edu/data/ectools). All datasets were downsampled to 100X before assembly.

The Oxford Nanopore and Illumina data for *Bacillus anthracis* Sterne 34F2 (PRJNA357857) and *Yersinia pestis* 195/P (PRJNA357858) sequencing data can be accessed from NCBI. The Illumina data was trimmed with Trimmomatic and only the surviving paired-end sequences were used for analysis:

```
java -jartrimmomatic-0.36.jar  PE -phred33 input.r1.fastq.gz input.r2.fastq.gz output.r1.fastq
output.r1.bad.fastq output.r2.fastq output.r2.bad.fastq ILLUMINACLIP:NexteraPE-PE.fa:2:30:10
LEADING:3 TRAILING:3 SLIDINGWINDOW:4:15 MINLEN:36
```

PacBio SMRT sequencing was downloaded from DevNet (https://github.com/PacificBiosciences/DevNet/wiki/Datasets) and is available from NCBI SRA. *E. coli* K12 (https://s3.amazonaws.com/files.pacb.com/datasets/secondary-analysis/e-coli-k12-P6C4/p6c4_ecoli_RSII_DDR2_with_15kb_cut_E01_1.tar.gz), *C. elegans* (http://datasets.pacb.com.s3.amazonaws.com/2014/c_elegans/list.html), *A. thaliana* (SRX533607), *D. melanogaster* (SRX499318), *H. sapiens* (PRJNA246220), and diploid *H. sapiens* HX1 (PRJNA301527). In all cases the raw H5 files and fastq sequences were downloaded. Fastq sequences were used for assembly.

Oxford Nanopore data from Loman et al. (Loman et al. 2015) was downloaded from EBI (ERX708228, ERX708229, ERX708230, ERX708231) sequencing *E. coli* K12 MG1665 and co-assembled as one dataset (MAP005). Four additional runs were generated for *E. coli* K12 by the same lab and available at (http://lab.loman.net/2015/09/24/first-sqk-map-006-experiment/). Raw sequences were downloaded from EBI (ERR1147227 (MAP006-1), ERR1147228 (MAP006-2), ERR1147229 (MAP006-PCR-1), ERR1147230 (MAP006-PCR-2) and assembled individually.

Supplemental Note 3: Repeat separation simulation

An anonymous reviewer suggested a test case using a simulated genome with two repeat copies. The mock genome is illustrated below:

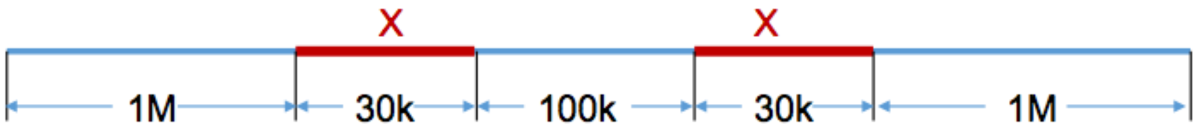

Multiple genomes were simulated with the second repeat copy mutated via single-nucleotide substitutions to have between 0% and 15% divergence. For each reference genome, reads were simulated using DAZZ DB to simulate reads at 12% error:

```
fasta2DAM dam test_reference.fasta
simulator dam -e0.12 -Mtest_repeat_reads.layout > test_repeat_reads.fasta
```

The defaults for read length ensured the longest sequences were <30Kb so the repeat would not be spanned. We ran all assemblers with default parameters. In addition to defaults, the Canu source code was modified to disable automatic error rate estimation and filtering within Bogart for a naïve comparison. As expected, no assembler could resolve the repeat copies when they were identical, and the Canu read graph showed the expected structure:

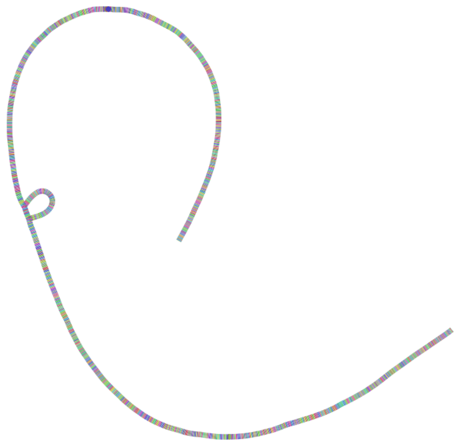

The repeat was considered resolved when an assembler produced an assembly with a single contig >2Mbp aligning to the reference. For Canu, the repeat was correctly resolved when the secondy copy was diverged by 3% or higher (Table S2).

Table S2: Assembly results for the two-copy repeat example

Divergance	Canu #ctg	Falcon #ctg	Miniasm #ctg	Naïve #ctg
0%	2	3	6	2
1%	2	3	3	2
2%	2	3	4	2
3%	(1)	3	3	2
4%	(1)	3	3	2
5%	(1)	(1)	3	2
6%	(1)	(1)	3	2
7%	(1)	(1)	2	2
8%	(1)	(1)	8	(1)
9%	(1)	(1)	5	(1)
10%	(1)	(1)	3	(1)
11%	(1)	(1)	5	(1)
12%	(1)	(1)	3	(1)
13%	(1)	(1)	(1)	(1)
14%	(1)	(1)	(1)	(1)
15%	(1)	(1)	(1)	(1)

Resolved genomes denoted by (1)

Supplemental Note 4: Low Coverage Assembly

SPAdes 100X+20X and SPAdes 100X+150X contained 79,651 and 79,025 contigs, respectively. Only contigs greater than 1,000 bp (2,696 and 2,428 respectively) were included for the analysis. The filtered contig set did not decrease the percentage of the reference covered by the full set, indicating the shorter contigs are likely redundant or assembly artifacts. SPAdes 100X+20X has a smaller max and NG50 than Canu but a higher accuracy than the initial assembly. Polishing with Quiver or Pilon increases the QV to one that is comparable with SPAdes. Despite having a higher max contig, the SPAdes 100X+150X NG50 is almost equivalent to Canu with comparable error statistics, despite having over 10-fold more sequencing data.

Table S3: Continuity and QV statistics *for A. thaliana* hybrid vs. hierarchical assemblies

Assembler	Assembly					Polishing				
	Max (Mb)	N50 (Mb)	Breaks	% ref covered	QV	Max (Mb)	N50 (Mb)	Breaks	% ref covered	QV
Canu 20X +Quiver	4.54	1.16	185	79.07%	19.23	4.54	1.17	183	81.83%	20.11
Canu 20X +Pilon 100X	4.54	1.16	185	79.07%	19.23	4.54	1.16	201	81.91%	20.31
SPAdes 100X +20X+Pilon 100X	3.71	0.84	156	82.23%	20.32	3.71	0.84	155	82.24%	20.32
SPAdes 100X +150X+Pilon 100X	5.69	1.23	163	82.40%	20.32	5.69	1.23	164	82.43%	20.32

Figure S1: Canu 20X+Quiver, Canu 20X+Pilon, and SPAdes 100X+20X and SPAdes 100X+150X *A. thaliana* assemblies
The plot shows the best (1-to-1) alignments between the reference (x-axis) and each assembly (y-axis). Red lines indicate forward-strand matches while blue lines indicate reverse-complement matches. Dashed vertical lines delineate chromosome ends while dashed horizontal lines delineate contigs. A diagonal indicates concordant matches while off-diagonal matches indicate assembly errors or differences versus the reference.

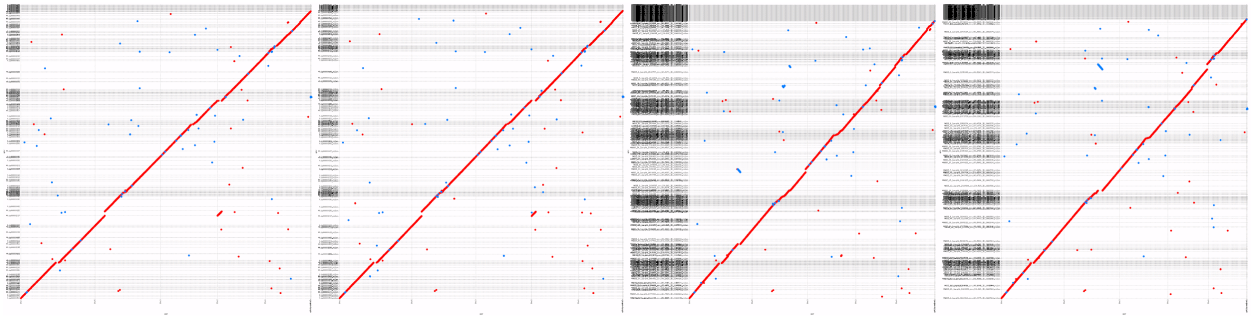

Supplemental Note 5: Assemblers

Falcon

Falcon v0.4.1 was checked out on 2016-03-16 (commit `c602aad3667b3fd49263028dac44da8e42caa17c`). Each test was run using the configurations provided in examples, when available. The LG configuration was used for CHM1. No configuration was available for *C. elegans* so the same one as *D. melanogaster* was used. For Oxford Nanopore datasets, read names were altered using a script to match DALIGNER expectation (https://github.com/jts/nanocorrect/blob/master/nanocorrect-preprocess.pl).

SPAdes

SPAdes v3.7.1 was used for all experiments and run for Nanopore datasets using:

```
spades.py -t 48 -m 128 -o asm -1 <illumina.1.fastq> -2 <illumina.2.fastq> --nanopore
<ont.fasta>
```

and for PacBio datasets using:

```
spades.py -t 48 -m 128 -o asm -1 <illumina.1.fastq> -2 <illumina.2.fastq> --pacbio <pac.fasta>
```

Miniasm

Minimap/miniasm was checked out of github on 2016-03-16 (commit `1cd6ae3bc7c7a6f9e7c03c0b7a93a12647bba244` minimap, `17d5bd12290e0e8a48a5df5afaeaef4d171aa133` miniasm). Minimap/miniasm ran as specified in the `misc/demo-worm-pacbio.sh` script with the commands:

```
minimap -k 15 -Sw5 -L100 -m0 -t48 -I6G reads.fasta reads.fasta |gzip -1 > reads.paf.gz
miniasm -f reads.fasta reads.paf.gz > reads.gfa
```

GFA primary sequences were converted to a fasta file for downstream analysis.

Canu

Canu version 1.3 was used for all experiments. The default Canu error rates, 0.05 for Oxford Nanopore data and 0.025 for PacBio data, are designed to work on a variety of datasets. The error rate is an upper bound on overlaps used in assembly and runtime can be improved by decreasing it. MAP005 *E. coli* ran with the default parameters. Newer Oxford Nanopore datasets, MAP006 *E. coli* datasets and *Y. pestis,* which are more similar to PacBio sequence error rates, ran with errorRate=0.025. *B. anthracis* used sensitive parameters (corMhapSensitivity=high corOutCoverage=80 errorRate=0.04). The *Y. pestis* had one high-coverage unassembled unitig with 15 reads which was included with the assembled contigs for downstream analysis and polishing. PacBio experiments used errorRate=0.013, as suggested for high-coverage PacBio datasets on the online FAQ (http://canu.readthedocs.io/en/latest/faq.html), matching the corrected read error rate used by Falcon.

Supplemental Note 6: Polishing

For PacBio data raw H5 bax.h5 were input to Quiver from SMRTportal 2.3.0 patch4 using the SGE pipeline available at https://github.com/skoren/QuiverGrid.

For Oxford Nanopore data, two alternative polishing strategies were used. First using Nanopolish (Loman et al. 2015) checked out on 2016-03-16 (commit `aba1b3201f46b4a00ae7463da3d2e3142366fd0e`) using the SGE pipeline available at https://github.com/skoren/NanoGrid. Second, using complementary Illumina data, polishing was performed with Pilon 1.13 with the commands:

```
bwa index <asm.fasta>
bwa map <asm.fasta> <illumina.1.fastq> <illumina.2.fastq>
java -jar pilon.jar --fix bases,local --genome <asm.fasta> --output <asm.pilon.fasta> --
changes --vcf --diploid
```

using the SGE pipeline available at https://github.com/skoren/PilonGrid.

Supplemental Note 7: Validation

Validation was perfomed using MUMer3.23 (Kurtz et al. 2004) and dnadiff (Phillippy et al. 2008). For *E. coli, B. anthracis, and Y. pestis* the command:

```
dnadiff reference.fasta asm.fasta
```

For the larger genomes to improve runtime, the commands were:

```
nucmer -l 100 -c 1000 reference.fasta asm.fasta
dnadiff -d out.delta
```

For the unpolished 1D *E. coli* and the 1D+2D *S. cerevisae* assemblies reference alignments were run with relaxed MUMmer parameters, to identify low-identity matches, for both Canu and Miniasm:

```
nucmer -l 10 -c 100 reference.fasta
dnadiff -d out.delta
```

Percent reference covered and identity was reported from 1-to-1 alignments and breakpoints were calculated as the sum of Relocations, Translocations, and Inversions. Validation was performed after assembly and after each round of polishing.

For *E. coli* K12 the reference used was NC_000913.3. For *B. anthracis* the reference was GCF_000008445.1. For *Y. pestis* the reference was GCF_000009065.1. For *D. melanogaster* the reference was Release 6 (GCF_000001215.4). For *A. thaliana* the reference was TAIR10 (GCF_000001735.3). For *C. elegans* the reference was GCF_000002985.6. for *H. sapiens,* the reference was GRCh38 (GCF_000001405.34).

Supplemental Note 8: Assembler Runtimes

All tests ran on an SGE grid composed of 24 hosts, each with dual Intel(R) Xeon(R) CPU E5-2680 v3 @ 2.50GHz CPUs (24-cores, 48 hyperthreads) and 128 GB of ram. Canu and Falcon were allowed to use SGE to submit processes to the grid using built-in grid management code. Since Miniasm and SPAdes are designed to run on a single shared-memory machines, each was allowed exclusive use of a machine.

Table S4: Canu assembly time (CPU hours)

Genome	Raw MHAP Index	Raw MHAP Overlapping	Read Correction	Trim overlapInCore Overlapping	Unitigging overlapInCore Overlapping	Total Assembly Time
E. coli	1.45	0.35	1.95	0.12	0.19	4.26
D. melanogaster	28.29	36.85	192.25	88.68	215.71	573.65
A. thaliana	46.32	116.65	80.90	151.74	157.87	577.49
C. elegans	17.99	9.11	36.30	14.64	88.22	170.14
CHM1	455.81	2340.66	1,624.57	6,945.50	6,401.53	18,019.70
MAP005	0.60	0.15	1.97	3.05	3.21	9.38
MAP006-1	0.80	0.17	2.09	0.55	1.00	4.85
MAP006-2	0.56	0.08	1.27	0.29	0.43	2.78
MAP006-PCR-1	0.57	0.52	1.47	0.12	0.19	3.04
B. anthracis	2.63	0.84	7.61	23.77	26.15	63.13
Y. pestis	1.59	0.76	3.38	1.07	5.53	12.55

Times were broken out to multiple steps, including the time to build MHAP indicies (Raw MHAP index), the time to compute overlaps given the index (Raw MHAP overlapping), time to compute alignments and generate corrected read consensus sequences (read correction), and time to compute correct read overlaps for both the trimming and unitigging Canu stages. In all cases for raw data, Canu computed the all-vs-all overlaps for the input raw data.

Table S5: Falcon assembly time (CPU hours)

Genome	Raw DALIGNER Overlapping	Read Correction	Corrected DALIGNER Overlapping	Total Assembly Time
E. coli	2.10	7.10	0.86	10.08
D. melanogaster	564.91	273.94	89.29	930.76
A. thaliana	654.47	140.18	39.92	836.61
C. elegans	70.55	99.21	32.68	202.74
CHM1	51,282.2	2,252.99	10,472.40	64,087.80
MAP005	0.18	2.63	0.16	2.98
MAP006-1	0.41	4.14	0.36	4.93
MAP006-2	0.14	1.31	0.11	1.56
MAP006-PCR-1	0.14	1.47	0.12	1.74
MAP006-PCR-2	0.53	6.44	0.43	7.42
B. anthracis	2.8	9.67	0.26	12.75
Y. pestis	0.48	3.86	0.22	4.57

Times were broken out as for Canu, with time to generate raw overlaps (raw DALIGNER overlapping), time to align and generate corrected consensus sequences (read correction), and time to overlap the corrected sequences. In all cases DALIGNER uses a seed read length threshold (set in the Falcon spec) to limit overlaps to only the longest sequences.

Table S6. Miniasm assembly time (CPU hours)

Genome	Raw minimap overlapping	Unitigging	Total Assembly Time
E. coli	0.17	0.01	0.18
D. melanogaster	19.03	0.44	19.47
A. thaliana	35.03	1.20	36.23
C. elegans	8.08	0.15	8.23
CHM1	3,109.20	N/A	N/A
MAP005	0.02	0.00	0.02
MAP006-1	0.04	0.01	0.05
MAP006-2	0.02	0.00	0.02
MAP006-PCR-1	0.02	0.00	0.02
MAP006-PCR-2	0.06	0.00	0.06
B. anthracis	0.1	0.01	0.11
Y. pestis	0.05	0.05	0.10

The time for both steps in Miniasm, overlapping (minimap) and assembly (unitigging with Miniasm) are reported. In all cases, minimap computed all-vs-all overlaps for the input raw data.

Table S7: SPAdes assembly time (CPU hours)

Genome	Total Assembly Time
E. coli	4.08
MAP005	3.61
MAP006-1	3.65
MAP006-2	3.56
MAP006-PCR-1	3.57
MAP006-PCR-2	4.00
B. anthracis	8.47
Y. pestis	17.08

Supplemental Note 9: PacBio Assembly Statistics with Quiver

Quiver (Chin et al. 2013) relies on the signal-level quality values from PacBio and a model trained on a specific chemistry to correct errors. The tables show quality for the initial assembly and after multiple rounds of Quiver. The figures show an alignment of the final assembly for each assembler to the reference genome. The final assembly is after one round of Quiver (for Canu and Falcon) and four rounds (for Miniasm).

Table S8: Quiver Statistics for Canu

	Assembly			Quiver1			Quiver2		
Genome	% ref covered	QV	Time	% ref covered	QV	Time	% ref covered	QV	Time
E. coli	100.00%	46.81	4.26	100%	58.90	7.99	100%	58.90	7.54
D. melanogaster	97.22%	31.72	573.65	97.47%	36.88	822.87	97.48%	37.21	824.25
A. thaliana	82.90%	20.31	577.49	82.94%	20.32	347.82	82.93%	20.32	376.72
C. elegans	99.61%	33.46	170.14	99.70%	35.93	239.93	99.7%	35.76	229.93
CHM1	86.48%	25.14	18,019.70	86.84%	27.17	4,730.01	86.86%	27.19	4,796.51

Each assembly was aligned to the reference genome using MUMmer and errors identified using dnadiff (Supplemental Note 7). Summary assembly consensus accuracy statistics are reported for the initial assembly as well as multiple rounds of polishing. QV was computed from total SNPs versus the reference and the total aligned bases in the assembly.

Table S9: Quiver Statistics For Falcon

	Assembly			Quiver1			Quiver2		
Genome	% ref covered	QV	Time	% ref covered	QV	Time	% ref covered	QV	Time
E. coli	100%	30.22	10.08	100%	58.22	7.67	100%	58.89	7.39
D. melanogaster	95.52%	27.14	930.76	96.12%	37.27	1375.16	96.15%	37.34	1359.23
A. thaliana	82.06%	19.87	836.61	82.72%	20.32	295.64	82.73%	20.32	335.51
C. elegans	98.70%	26.59	202.74	98.82%	35.76	194.66	98.82%	35.69	175.32
CHM1	86.03%	23.25	64,087.80	86.58%	27.18	4,701.18	86.62%	27.19	4,702.71

Same columns as Table S8.

Table S10: Quiver Statistics For Miniasm

	Assembly			Quiver1			Quiver2			Quiver3			Quiver4		
Genome	% ref covered	QV	Time	% ref covered	QV	Time	% ref covered	QV	Time	% ref covered	QV	Time	% ref covered	QV	Time
E. coli	95.20%	9.36	0.18	99.98%	37.67	8.19	99.99%	57.65	7.78	99.99%	57.12	8.38	99.99%	57.12	7.40
D. melanogaster	0.00%	0	19.47	96.42%	35.05	282.83	96.48%	36.95	383.80	96.50%	37.20	395.93	96.51%	37.27	402.30
A. thaliana	0.00%	0	36.23	82.79%	20.26	197.45	82.88%	20.32	255.95	82.87%	20.32	250.71	82.88%	20.32	236.09
C. elegans	0.00%	0	8.23	99.24%	32.14	111.35	99.37%	34.61	127.54	99.43%	35.14	133.16	99.44%	35.32	145.88

Same columns as Table S8.

Table S11: Quiver Assembly Statistics For SPAdes

	Assembly			Quiver1			Quiver2		
Genome	% ref covered	QV	Time	% ref covered	QV	Time	% ref covered	QV	Time
E. coli	100%	44.55	4.09	99.97%	47.97	9.82	100%	45.05	7.44

Same columns as Table S8.

Figure S2: Canu, Falcon, Miniasm, and SPAdes *E. coli* K12 polished assembles
See caption for Figure S1.

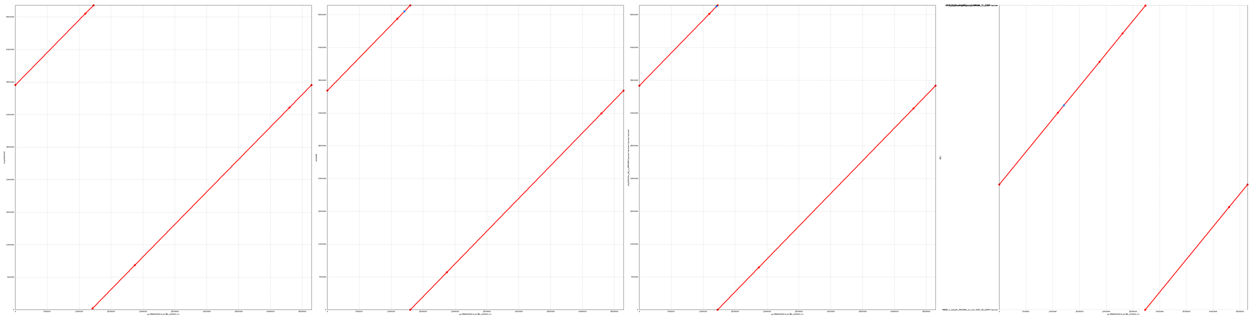

Figure S3: Canu, Falcon, and Miniasm *D. melanogaster* assemblies
See caption for Figure S1.

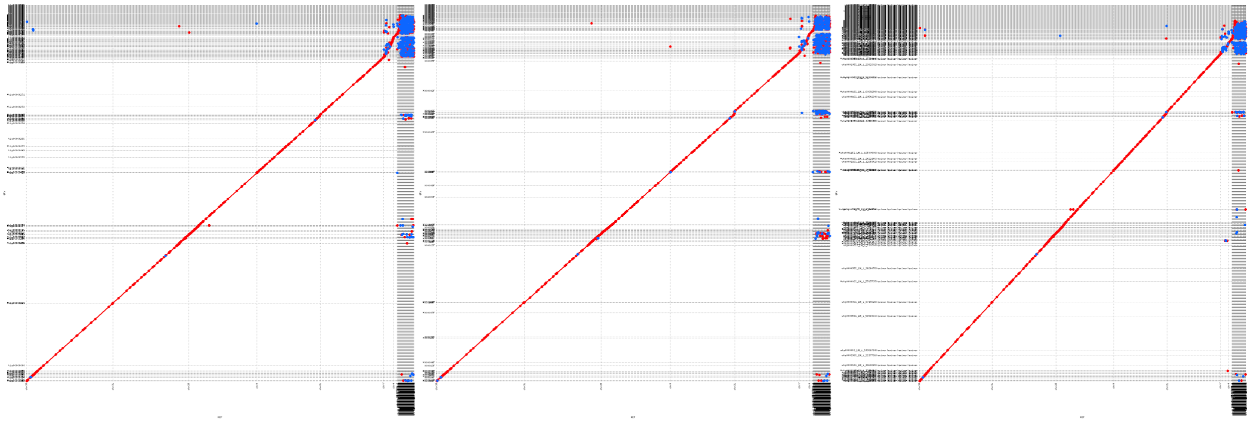

Figure S4: Canu, Falcon, and Miniasm *A. thaliana* assemblies
See caption for Figure S1.

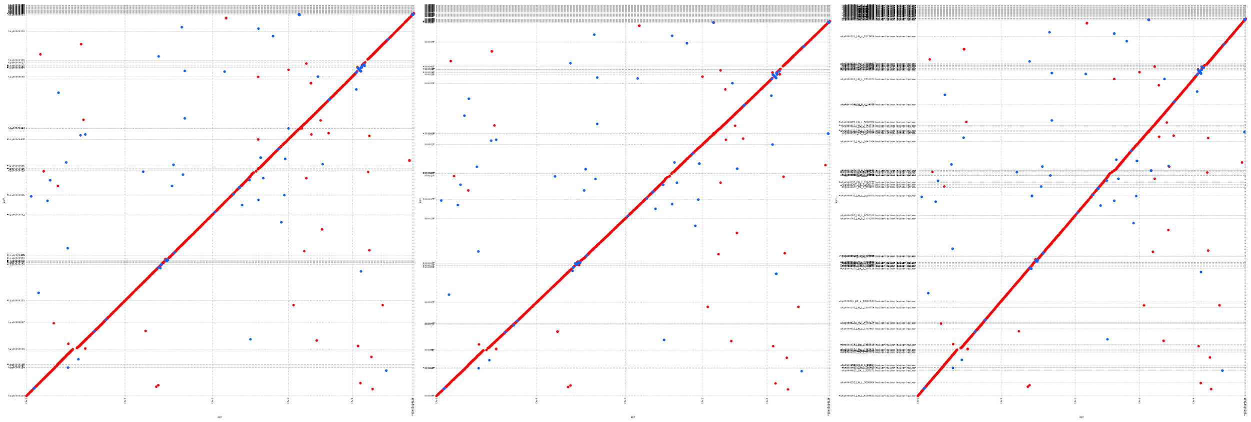

Figure S5: Canu, Falcon, and Miniasm *C. elegans* assemblies
See caption for Figure S1.

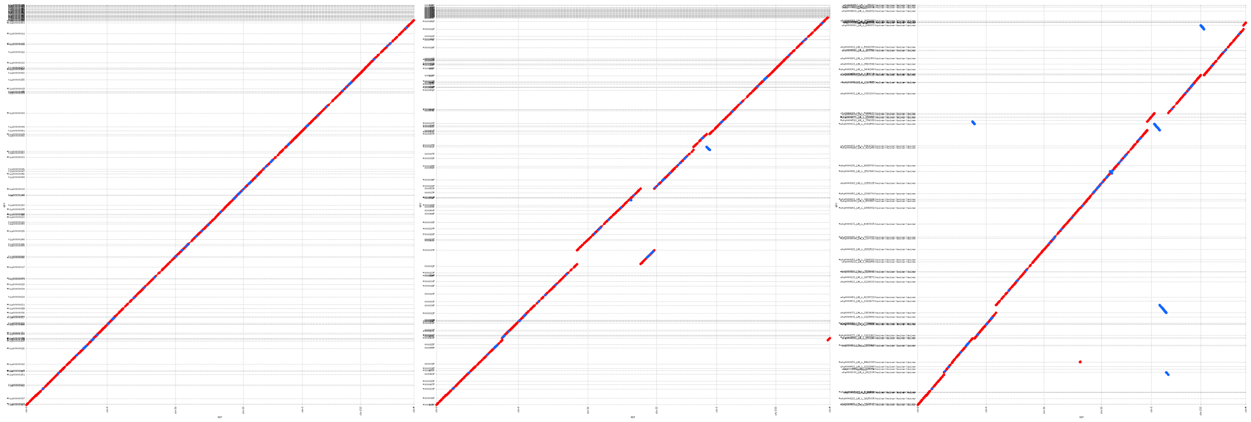

Figure S6: Canu and Falcon assemblies of *H. sapiens* CHM1
See caption for Figure S1.

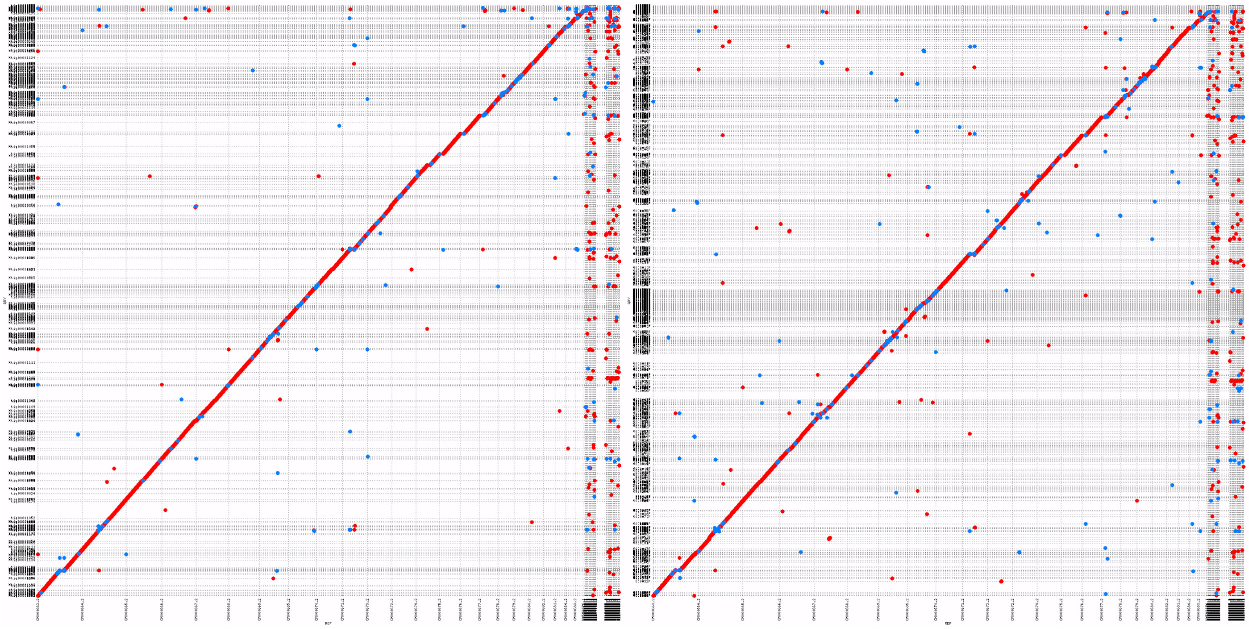

Figure S7: Read length distributions for CHM1 P5-C3 (left) and HX1 (right)
Only sequences longer than 500bp in both are shown.

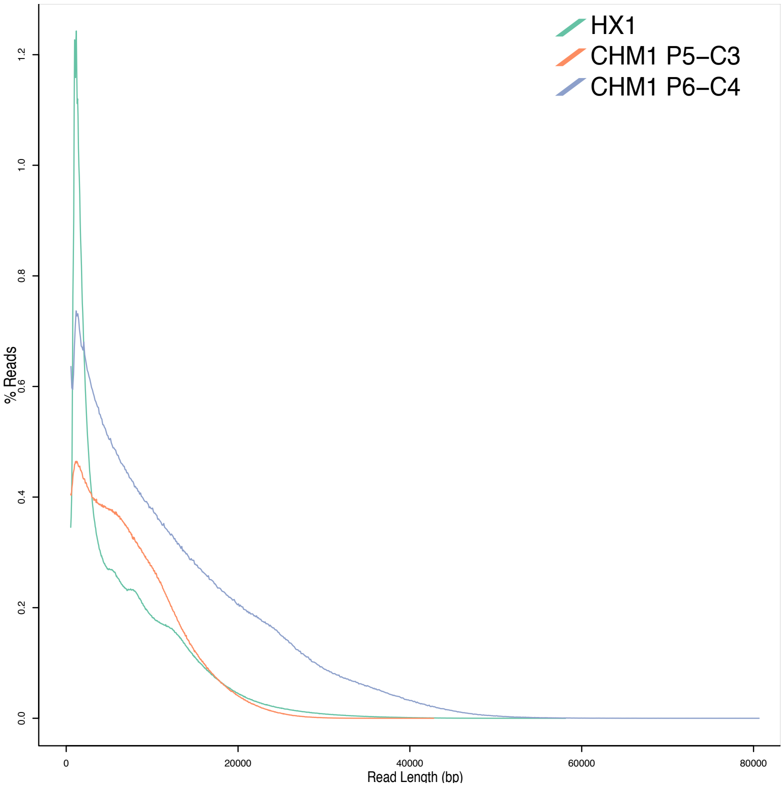

Figure S8: Canu and published assemblies of *H. sapiens* HX1
See caption for Figure S1.

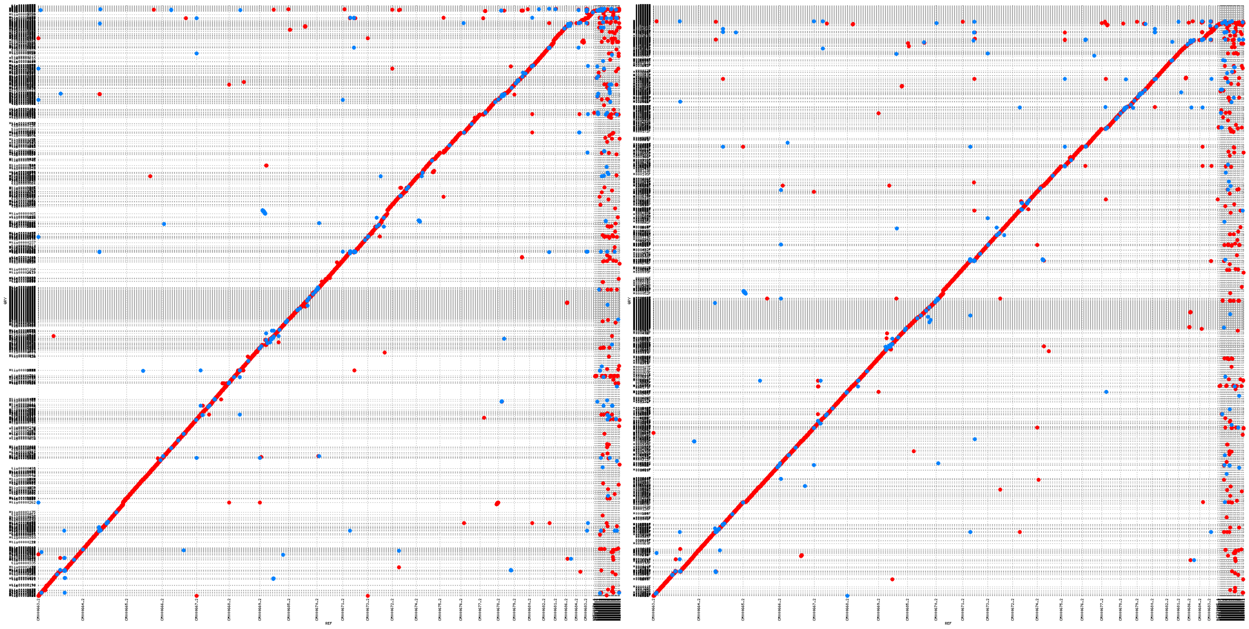

Supplemental Note 10: Oxford Nanopore Assemblies with Nanopolish

Nanopolish (Loman et al. 2015) relies on the events underlying the basecall for a Nanopore sequence and a model of the sequencing error to correct errors. The tables show quality for the initial assembly and after multiple rounds of Nanopolish. The figures show an alignment of the final assembly for each assembler to the reference genome. The final assembly is after one round of Nanopolish (for Canu and Falcon) and three rounds (for Miniasm).

Table S12: Nanopolish statistics for Canu

	Assembly			Nanopolish 1			Nanopolish 2		
Genome	% ref covered	QV	Time	% ref covered	QV	Time	% ref covered	QV	Time
MAP005	99.97%	14.59	9.38	99.98%	22.44	367.49	99.98%	22.91	202.82
MAP006-1	99.79%	21.53	4.85	99.80%	27.11	162.19	99.81%	27.19	154.01
MAP006-2	99.85%	20.91	2.78	99.91%	26.61	165.91	99.92%	26.86	151.57
MAP006-PCR-1	99.95%	22.28	3.04	99.95%	27.92	161.04	99.95%	27.93	142.63
MAP006-PCR-2	99.99%	22.42	5.25	99.99%	28.27	200.84	100.00%	28.41	171.34
B. anthracis	99.77%	15.69	63.13	99.77%	20.65	831.27	99.77%	21.04	468.11
Y. pestis	99.97%	21.35	12.55	99.97%	26.13	241.70	99.97%	26.20	192.00

Same columns as Table S8.

Table S13: Nanopolish statistics for Falcon

	Assembly			Nanopolish 1			Nanopolish 2		
Genome	% ref covered	QV	Time	% ref covered	QV	Time	% ref covered	QV	Time
MAP005	22.89%	13.54	2.98	23.03%	22.29	103.22	23.00%	23.07	38.55
MAP006-1	99.86%	19.38	4.93	99.86%	26.64	202.52	99.86%	27.16	174.34
MAP006-2	99.94%	18.68	1.56	99.94%	26.19	194.63	99.94%	26.89	153.68
MAP006-PCR-1	99.80%	20.16	1.74	99.80%	27.46	166.63	99.80%	27.88	143.49
MAP006-PCR-2	100.00%	20.31	7.42	100.00%	27.83	205.47	100.00%	28.35	179.51
B. anthracis	86.27%	14.73	12.75	86.29%	20.81	783.18	86.30%	21.41	368.58
Y. pestis	99.97%	19.16	4.57	99.97%	25.51	290.44	99.96%	25.95	188.91

Same columns as Table S8.

Table S14: Nanopolish statistics for Miniasm

	Assembly			Nanopolish 1			Nanopolish 2			Nanopolish 3		
Genome	% ref covered	QV	Time	% ref covered	QV	Time	% ref covered	QV	Time	% ref covered	QV	Time
MAP005	79.81%	7.81	0.02	99.62%	15.58	1801.51	99.93%	20.31	320.75	99.96%	21.94	221.74
MAP006-1	91.76%	9.24	0.05	99.71%	16.94	1363.27	99.94%	22.75	257.37	99.97%	25.56	180.33
MAP006-2	92.89%	9.18	0.02	99.48%	16.78	1130.5	99.68%	22.35	233.74	99.70%	25.15	118.69
MAP006-PCR-1	93.83%	9.52	0.02	99.79%	17.62	1014.35	99.93%	23.58	208.51	99.96%	26.28	115.40
MAP006-PCR-2	93.77%	9.31	0.06	99.89%	18.10	1457.47	99.97%	24.74	252.86	99.98%	27.18	159.24
B. anthracis	66.46%	7.75	0.11	97.07%	14.36	4013.98	97.19%	18.67	676.67	97.21%	20.23	404.14
Y. pestis	89.94%	9.20	0.10	99.65%	16.56	1502.52	99.90%	21.99	295.81	99.91%	24.61	201.73

Same columns as Table S8.

Figure S9: Canu, Falcon, and Miniasm assemblies of *E. coli* K12 MAP005.
See caption for Figure S1.

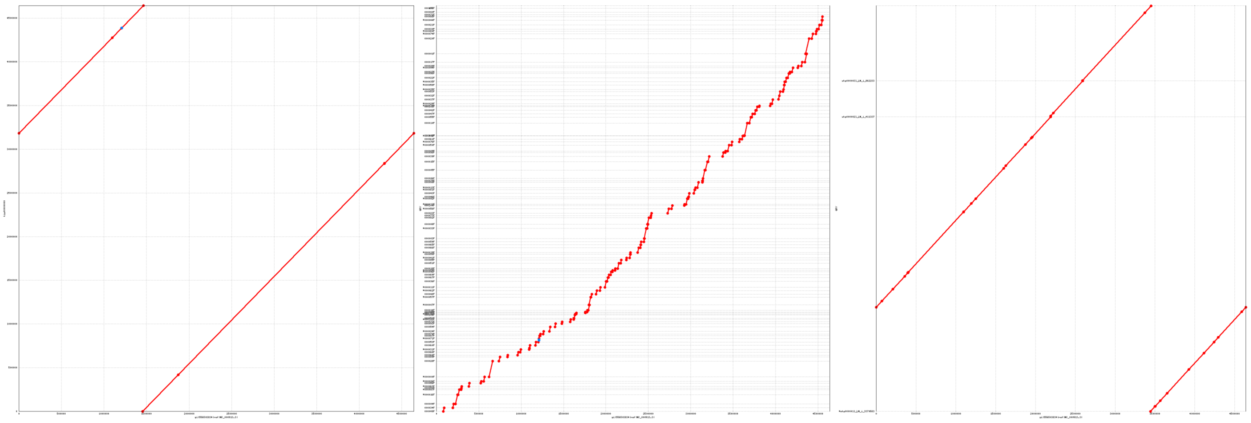

Figure S10: Canu, Falcon, and Miniasm assemblies of *E. coli* K12 MAP006-1.
See caption for Figure S1.

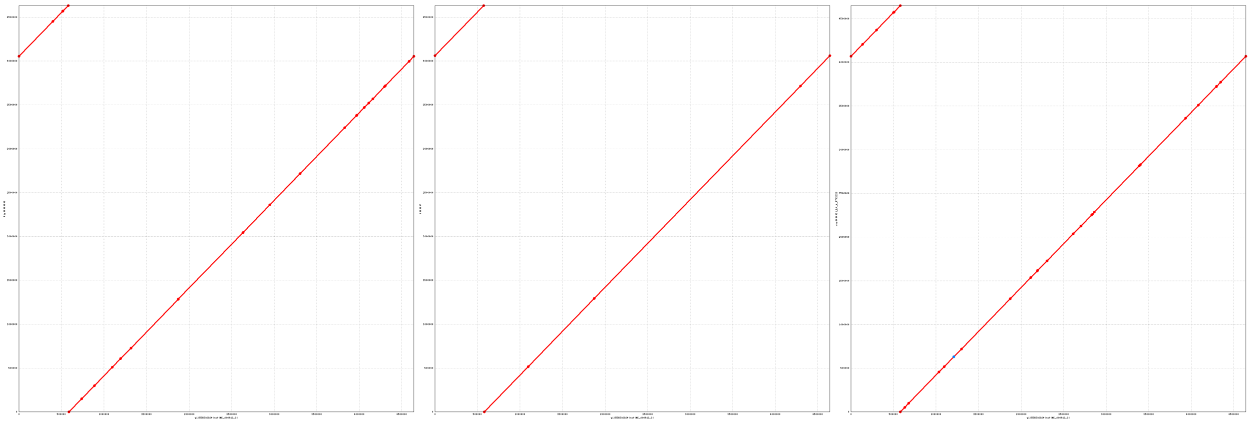

Figure S11: Canu, Falcon, and Miniasm assemblies of *E. coli* K12 MAP006-2.
See caption for Figure S1.

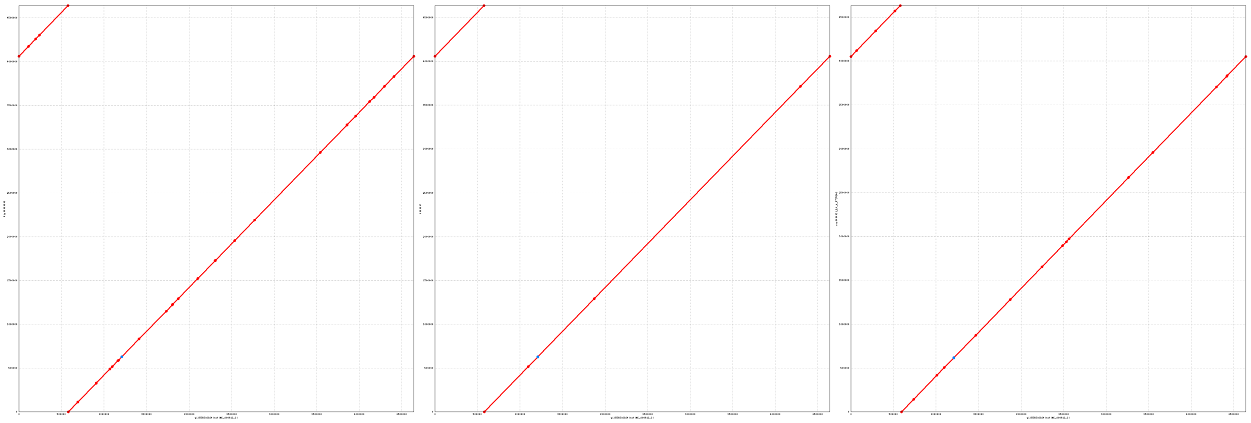

Figure S12: Canu, Falcon, and Miniasm assemblies of *E. coli* K12 MAP006-PCR-1.
See caption for Figure S1.

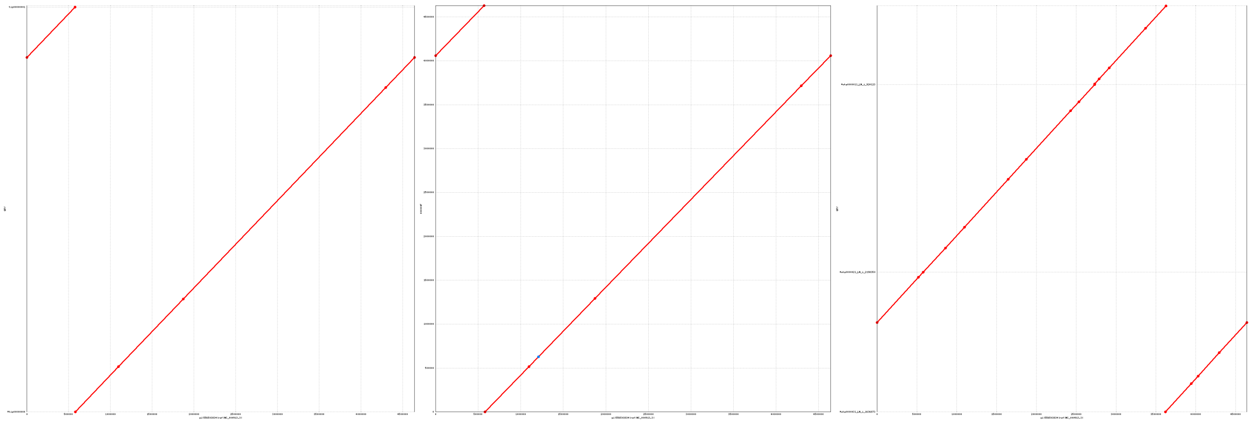

Figure S13: Canu, Falcon, and Miniasm assemblies of *E. coli* K12 MAP006-PCR-2.
See caption for Figure S1.

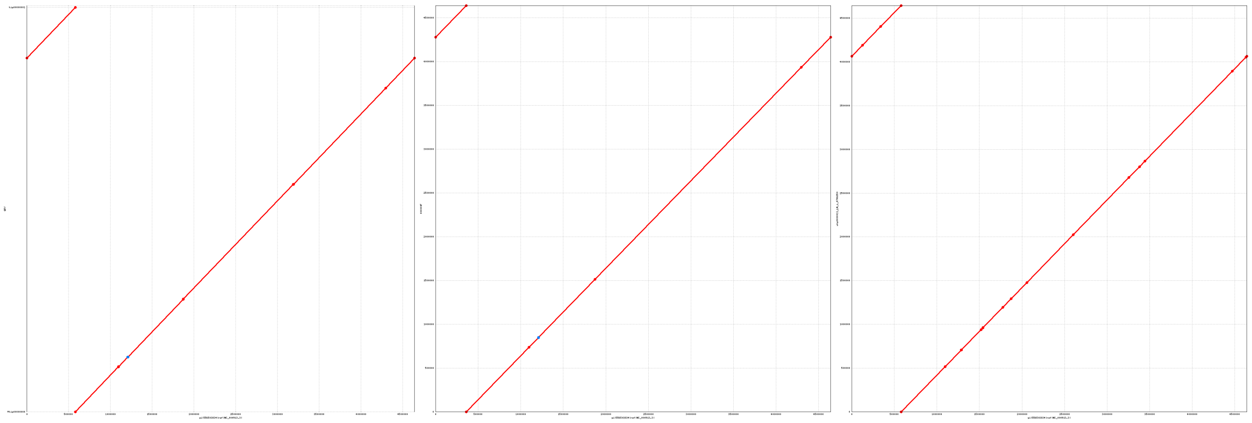

Figure S14: Canu, Falcon, and Miniasm assemblies of *B. anthracis*.
See caption for Figure S1.

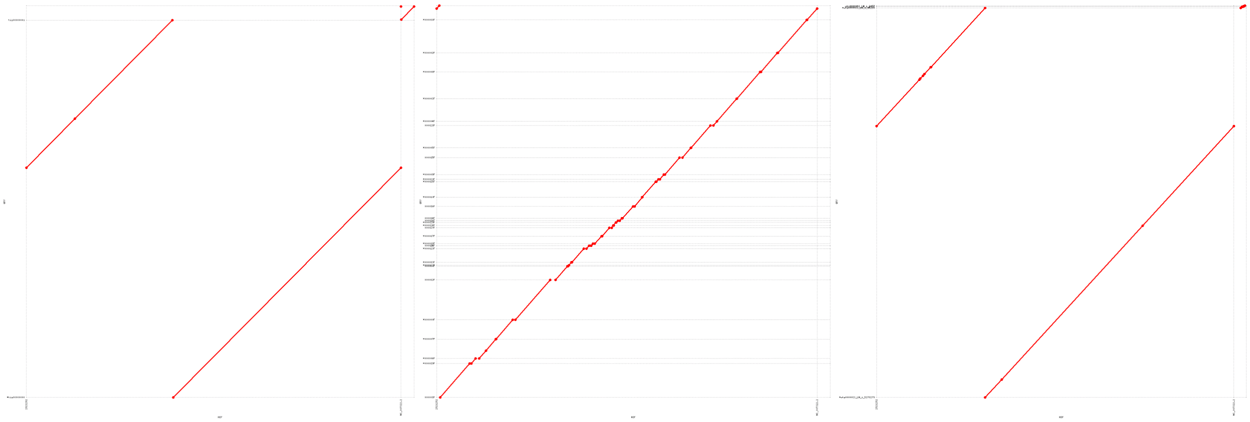

Figure S15: Canu, Falcon, and Miniasm assemblies of *Y. pestis*.
See caption for Figure S1.

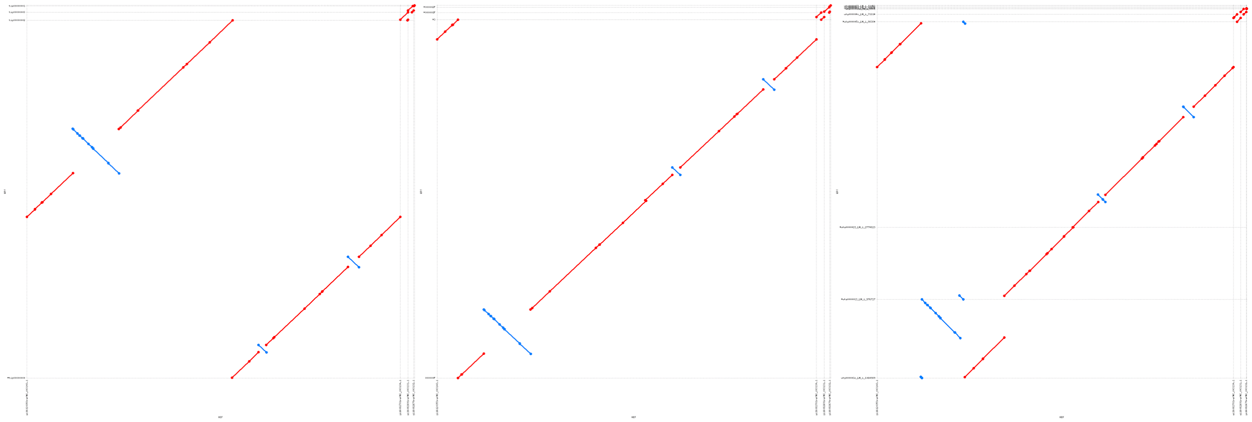

Supplemental Note 11: Oxford Nanopore assemblies with Pilon

Pilon (Walker et al. 2014) uses Illumina sequences aligned to an assembly to correct errors. The tables show quality for the initial assembly and after multiple rounds of Pilon. The figures show an alignment of the final assembly for each assembler to the reference genome. The final assembly is after three round of Pilon (for Canu, Falcon, and Miniasm) and unpolished (for SPAdes).

Table S15: Pilon statistics for Miniasm after 300 rounds of polishing

	Assembly			Pilon 300		
**Genome**	**% ref covered**	**QV**	**Time**	**% ref covered**	**QV**	**Total Time**
MAP005	79.81%	7.81	0.02	91.80%	16.79	182.57
MAP006-1	91.76%	9.24	0.05	97.07%	24.45	114.18
MAP006-2	92.89%	9.18	0.02	98.03%	24.55	96.82
MAP006-PCR-1	93.83%	9.52	0.02	98.46%	25.19	83.28
MAP006-PCR-2	93.77%	9.31	0.06	98.62%	25.18	94.96
B. anthracis	65.34%	7.75	0.11	79.88%	11.51	842.89
Y. pestis	89.94%	9.20	0.10	93.92%	19.49	543.77

Same columns as Table S8.

Figure S16: Minimap remaining errors in *E. coli* K12 MAP006-1

Using dnadiff after 300 rounds of pilon on miniasm we tabulated GAP entries in the rdiff file. These indicate unaligned regions between the reference and the assembly as well as the size of the discrepancy. The average discrepancy size was 569.17 bp in six discrepant regions. The figure highlights several gap regions which show a) an expansion in the assembly with respect to the reference b) a collapse in the reference with respect to the assembly.

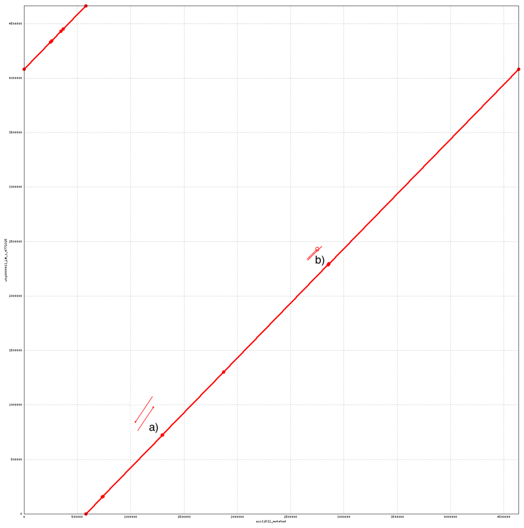

Table S16: Pilon statistics for Canu

	Assembly			Pilon 1			Pilon 2			Pilon 3		
Genome	% ref covered	QV	Time	% ref covered	QV	Time	% ref covered	QV	Time	% ref covered	QV	Time
MAP005	99.97%	14.59	9.38	99.97%	29.99	0.96	99.99%	38.62	0.32	99.99%	38.95	0.32
MAP006-1	99.79%	21.53	4.85	99.79%	40.98	0.50	99.82%	48.40	0.28	99.82%	53.44	0.26
MAP006-2	99.85%	20.91	2.78	99.89%	37.37	0.56	99.94%	42.90	0.29	99.94%	48.90	0.29
MAP006-PCR-1	99.95%	22.28	3.04	99.95%	43.72	0.56	99.95%	50.54	0.28	99.95%	51.75	0.27
MAP006-PCR-2	99.99%	22.42	5.25	100%	43.79	0.38	100%	50.44	0.27	100%	50.99	0.26
B. anthracis	99.77%	15.69	63.13	99.77%	25.56	0.83	99.77%	27.95	0.53	99.77%	28.17	0.52
Y. pestis	99.97%	21.35	12.55	99.87%	28.78	2.26	99.83%	29.62	1.52	99.83%	29.77	1.59

Same columns as Table S8.

Table S17: Pilon statistics for Falcon

	Assembly			Pilon 1			Pilon 2			Pilon 3		
Genome	% ref covered	QV	Time	% ref covered	QV	Time	% ref covered	QV	Time	% ref covered	QV	Time
MAP005	22.89%	13.54	2.98	23.06%	27.72	0.65	23.07%	33.31	0.41	23.07%	33.47	0.32
MAP006-1	99.86%	19.38	4.93	99.86%	33.77	1.30	99.86%	43.04	0.57	99.86%	44.43	0.50
MAP006-2	99.94%	18.68	1.56	99.94%	32.67	1.27	99.94%	40.24	0.58	99.94%	41.77	0.52
MAP006-PCR-1	99.80%	20.16	1.74	99.80%	35.44	0.76	99.80%	43.31	0.54	99.80%	45.13	0.51
MAP006-PCR-2	100%	20.31	7.42	100%	35.80	0.75	100%	42.17	0.54	100%	44.33	0.51
B. anthracis	86.27%	14.73	12.75	86.31%	24.95	0.45	86.31%	29.08	0.19	86.31%	29.54	1.47
Y. pestis	99.97%	19.16	4.57	99.71%	26.94	1.54	99.65%	28.73	2.74	99.65%	28.91	1.78

Same columns as Table S8.

Table S18: Pilon statistics for Miniasm

	Assembly			Pilon 1			Pilon 2			Pilon 3		
Genome	% ref covered	QV	Time	% ref covered	QV	Time	% ref covered	QV	Time	% ref covered	QV	Time
MAP005	79.81%	7.81	0.02	85.62%	10.87	1.43	88.46%	13.97	1.01	90.63%	15.83	0.69
MAP006-1	91.76%	9.24	0.05	93.09%	16.42	2.01	96.32%	22.23	0.74	96.97%	24.11	0.34
MAP006-2	92.89%	9.18	0.02	94.12%	16.05	1.87	97.47%	22.75	0.50	97.98%	24.36	0.34
MAP006-PCR-1	93.83%	9.52	0.02	95.17%	17.02	1.36	97.93%	23.20	0.49	98.41%	24.86	0.28
MAP006-PCR-2	93.77%	9.31	0.06	94.85%	16.45	1.83	98.15%	23.20	0.46	98.57%	24.86	0.34
B. anthracis	66.46%	7.75	0.11	77.41%	9.38	0.29	78.54%	10.45	2.19	79.36%	11.12	2.31
Y. pestis	89.94%	9.20	0.10	91.89%	14.09	2.18	93.16%	18.05	3.99	93.76%	19.16	2.41

Same columns as Table S8.

Table S19: Pilon statistics for Spades

	Assembly			Pilon 1			Pilon 2			Pilon 3		
Genome	% ref covered	QV	Time	% ref covered	QV	Time	% ref covered	QV	Time	% ref covered	QV	Time
MAP005	100%	44.55	3.61	100%	48.75	0.33	100%	49.27	0.37	100%	49.59	0.29
MAP006-1	100%	44.55	3.65	100%	48.74	0.39	100%	49.26	0.46	100%	49.59	0.30
MAP006-2	100%	44.55	3.56	100%	48.74	0.36	100%	49.26	0.28	100%	49.59	0.28
MAP006-PCR-1	100%	44.60	3.57	100%	48.54	0.28	100%	49.03	0.28	100%	49.34	0.27
MAP006-PCR-2	100%	44.60	4.00	100%	48.74	0.31	100%	49.26	0.29	100%	49.59	0.27
B. anthracis	100%	42.83	8.47	100%	44.82	1.06	100%	45.20	0.99	100%	45.28	0.90
Y. pestis	95.99%	33.56	17.08	96.00%	33.51	1.29	96.00%	33.53	1.29	96.00%	33.53	1.24

Same columns as Table S8.

Figure S17: Canu, Falcon, Miniasm, and SPAdes assemblies of *E. coli* K12 MAP005
See caption for Figure S1.

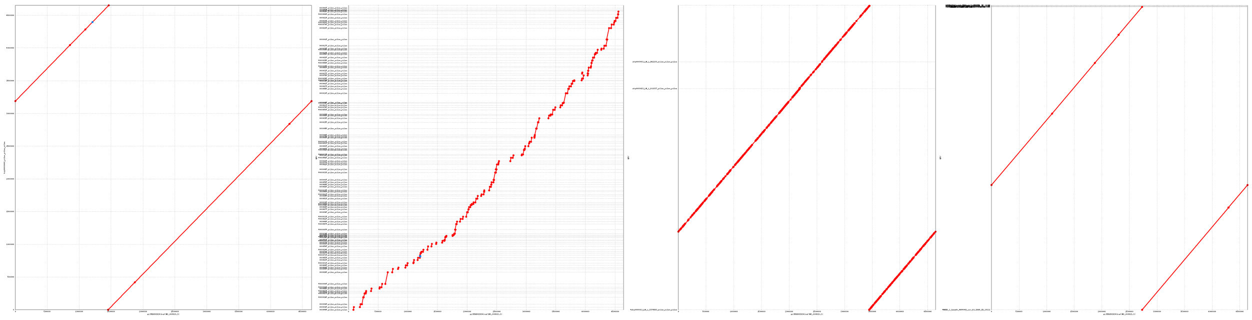

Figure S18: Canu, Falcon, Miniasm, and SPAdes assemblies of *E. coli* K12 MAP006-1
See caption for Figure S1.

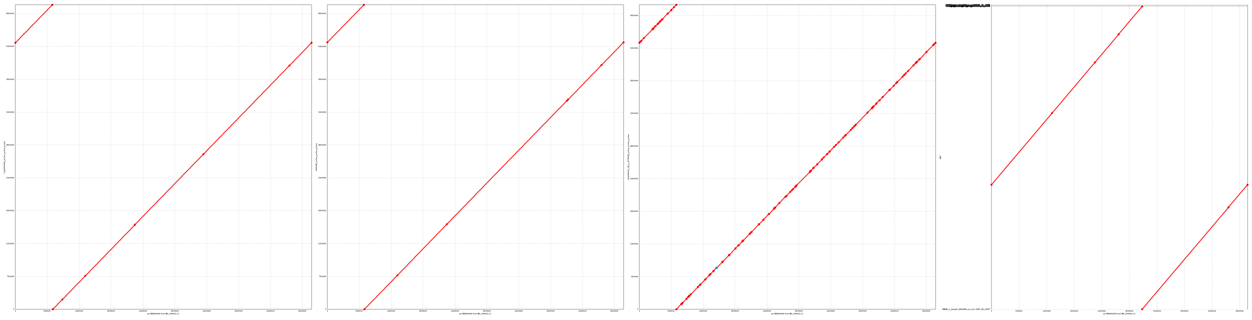

Figure S19: Canu, Falcon, Miniasm, and SPAdes assemblies of *E. coli* K12 MAP006-2
See caption for Figure S1.

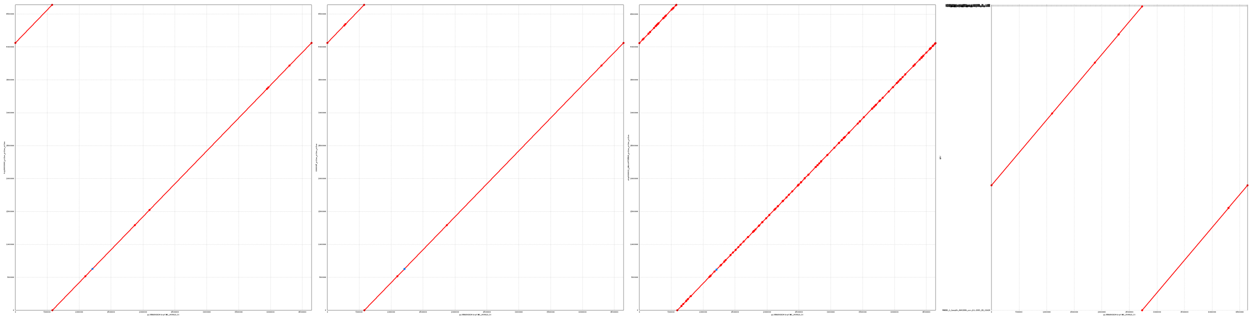

Figure S20: Canu, Falcon, Miniasm, and SPAdes assemblies of *E. coli* K12 MAP006-PCR-1
See caption for Figure S1.

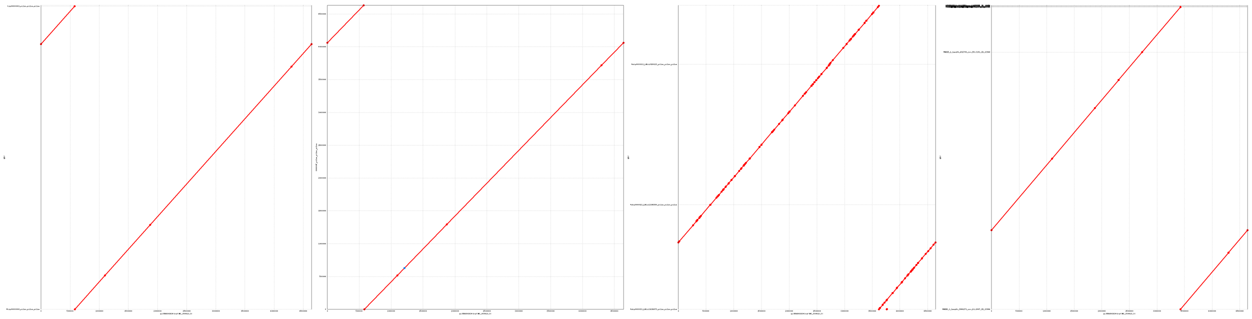

Figure S21: Canu, Falcon, Miniasm, and SPAdes assemblies of *E. coli* K12 MAP006-PCR-2
See caption for Figure S1.

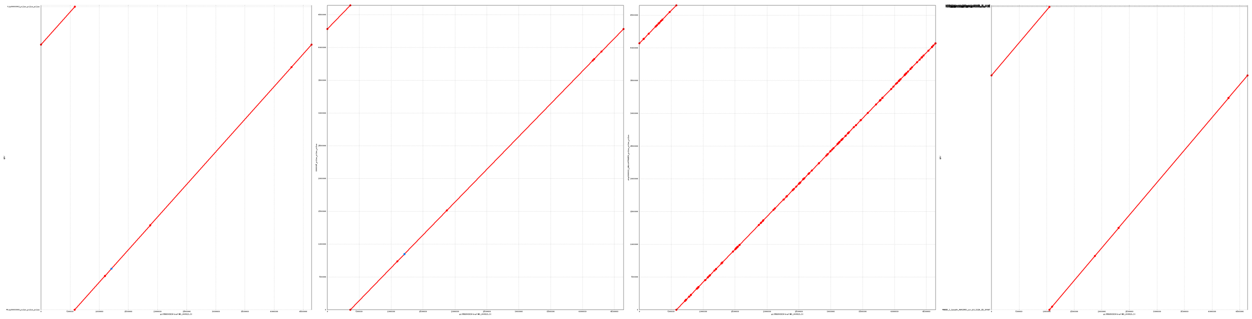

Figure S22: Canu, Falcon, Miniasm, and SPAdes assemblies of *B. anthracis*.
See caption for Figure S1.

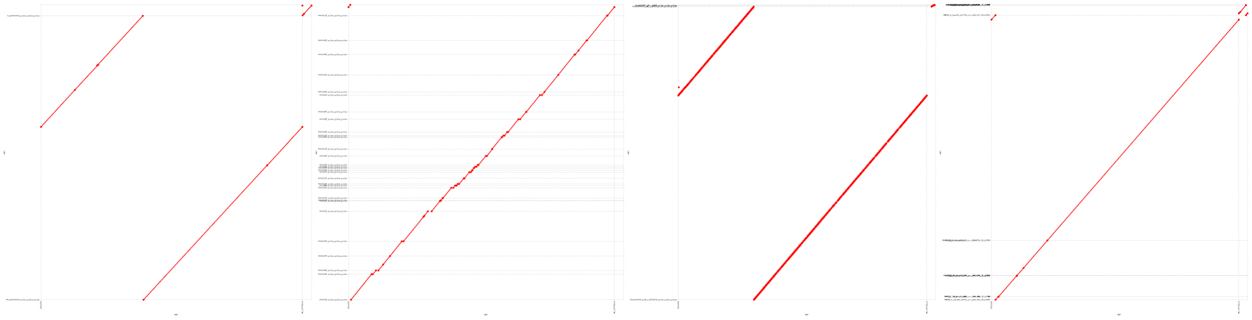

Figure S23: Canu, Falcon, Miniasm, and SPAdes assemblies of *Y. pestis*.
See caption for Figure S1.

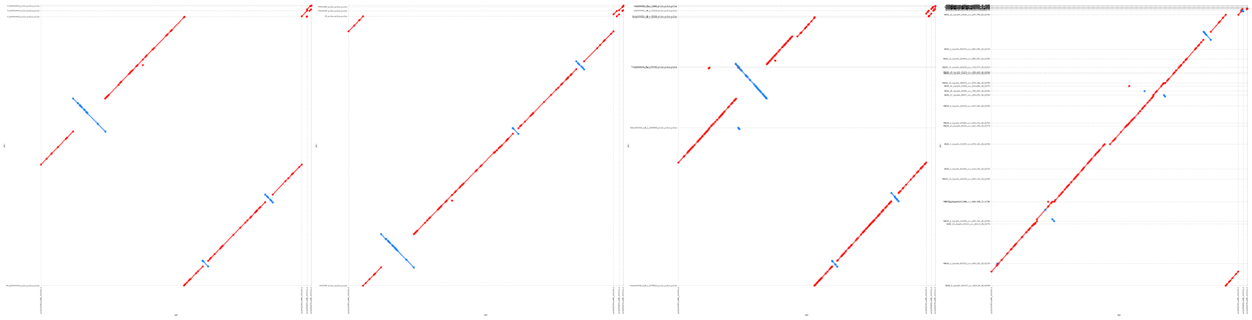

Supplemental Note 12: 1D Assembly

We ran 10 rounds of correction followed by assembly. For 1D:

```
NAME=1d.fasta
COUNT=0
   for i in `seq 1 10`; do
     canu -correct -p asm -d round$i \
          corOutCoverage=500 corMinCoverage=0 corMhapSensitivity=high \
          genomeSize=4.8m -nanopore-raw $NAME
      NAME="round$i/asm.correctedReads.fasta.gz"
      COUNT=`expr $COUNT + 1`
   done
   canu -p asm -d asm genomeSize=4.8m -nanopore-corrected $NAME \
        errorRate=0.1 utgGraphDeviation=50 batOptions="-ca 500 -cp 50"
```

The initial Canu assembly had 0 structural errors, covering 89.38% of the reference at 85.52% identity. We ran Nanopolish 1d-workflows with modifications available from https://github.com/skoren/nanopolish. Compiler optimization had to be disabled for Nanopolish to run to completion. We ran a total of 10 rounds of polishing, resulting in 96.67%, 97.78%, 98.04%, 98.18%, 98.25%, 98.31%, 98.34%, 98.37%, 98.38%, and 98.40% identity. Post three rounds of polishing the assembly has 1 errors, covering 93.32% of the reference at 98.04% identity. Post three Pilon polishing rounds it has 10 errors (all relocations due to collapsed sequences which could not be mapped pre-polishing), covering 93.84% of the reference at 99.70% identity. The initial Minimap assembly had 3 errors, covering only 9.19% of the reference at 75.76% identity.

Figure S24: Canu 1D, Canu+three rounds of Nanopolish, Canu 1D+three rounds of Pilon, and Miniasm assemblies for *E. coli* K12 MAP006-1 1D.
See caption for Figure S1.

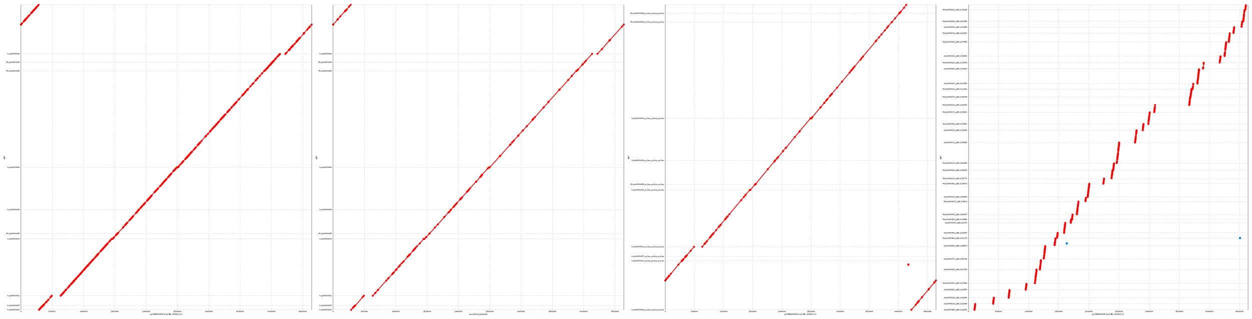

Supplemental Note 13: *S. cerevisae* Nanopore assembly

As above, we ran 10 rounds of correction followed by assembly for *S. cerevisae*:

```
  COUNT=0
   NAME=input.fasta
   for i in `seq 1 10`; do
```

```
      canu -correct -p asm -d round$i \
           corOutCoverage=500 corMinCoverage=0 corMhapSensitivity=high \
           genomeSize=12.1m -nanopore-raw $NAME
      NAME="round$i/asm.correctedReads.fasta.gz"
      COUNT=`expr $COUNT + 1`
   done
  canu -p asm -d asm genomeSize=12.1m -nanopore-corrected $NAME utgGraphDeviation=50
batOptions=”-ca 500 -cp 50”
done
```

The initial Canu assembly had 2 structural errors, covering 94.93% of the reference at 94.38% identity. Post polishing it has 14 errors, covering 96.86% of the reference at 99.83% identity. The reference (S288c) is not identical to the sequenced strain (W303) and the number of errors is similar to that from a high-coverage PacBio only assembly (29 ctg, 99.12% of ref, 99.88% idy, 13 errors) and could be true variation between the strains.

Figure S25: Canu 1D, and Canu 1D+three rounds of Pilon, and Canu PacBio 115X assemblies.
See caption for Figure S1.

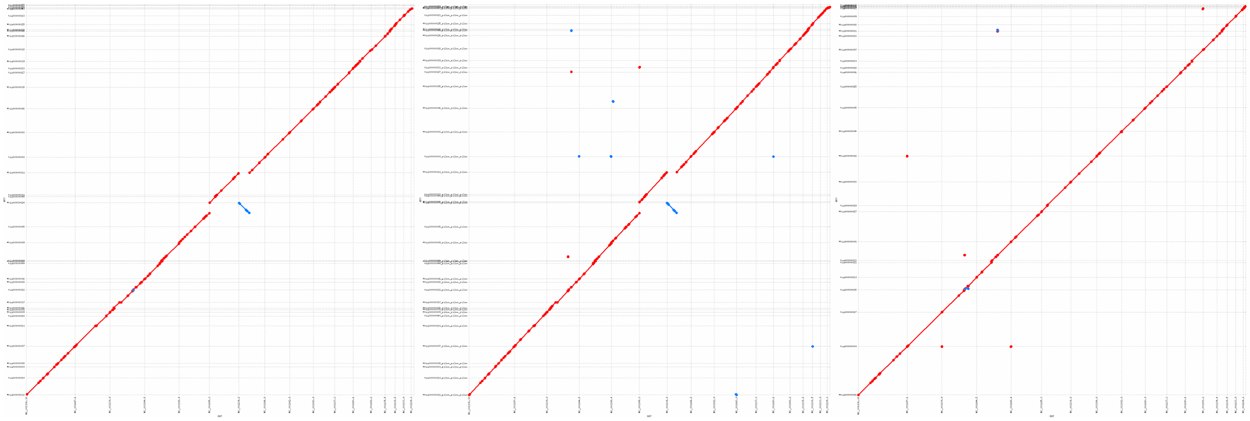

Figure S26: Parallel overlap store construction
Canu constructs the overlap store by a parallel bucket sort. Each overlap file is first bucketized based on their read range, with each bucket sized to be approximately the same size. Once bucketized, parallel processes collect all overlaps from a bucket and sort them independently. Finally, a merged index is written to disk.

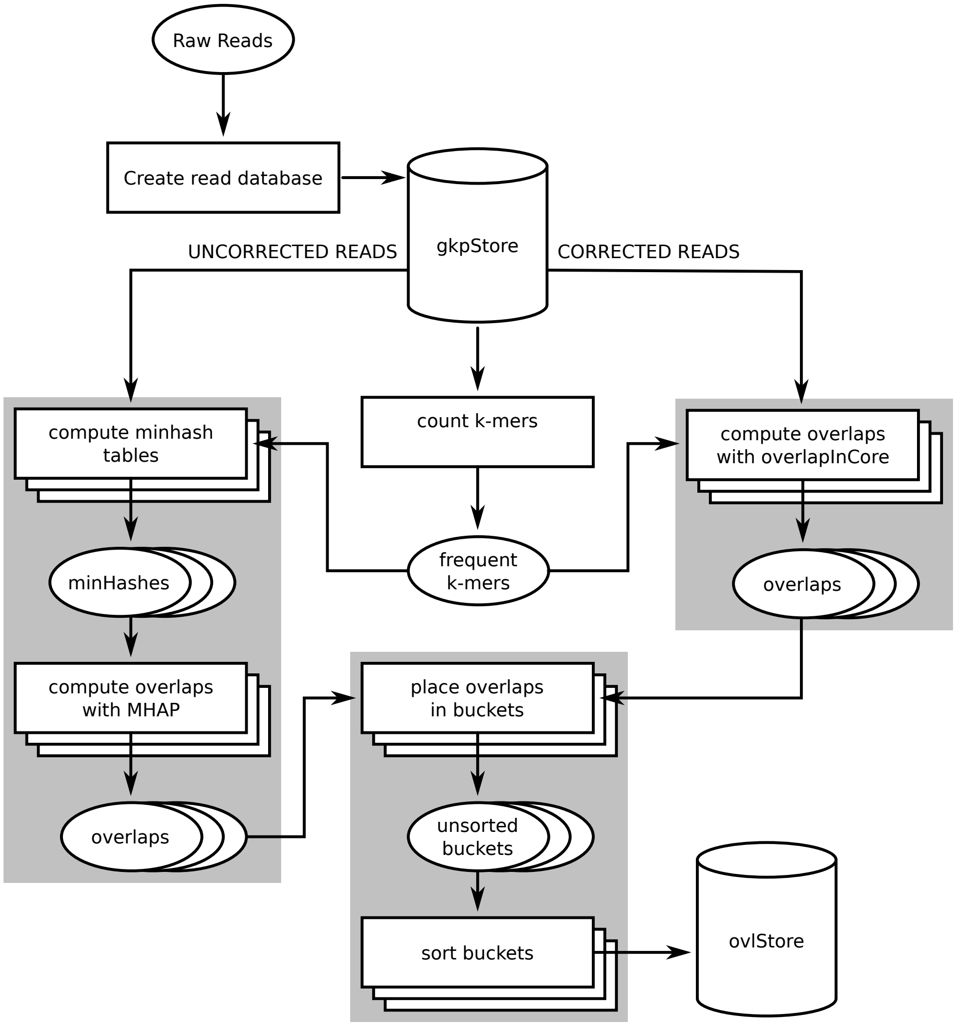

Figure S27: Suspicious read detection
A read *A* has an anomaly at position *X* which prevents the *A*-*C* overlap from being discovered. Read *A*'s best overlap is to read *B*, but read *B*'s best overlap is to read *C*. If read *A* ends in a repeat, the best overlap could be to a diverged repeat, leading to a misassembly.

```
A  -------------X------------

C       ----------------------------

B                 ----------------------------
```

References

Berlin K, Koren S, Chin C-S, Drake JP, Landolin JM, Phillippy AM. 2015. Assembling large genomes with single-molecule sequencing and locality-sensitive hashing. *Nat Biotech* **33**: 623-630.

Chaisson MJ, Tesler G. 2012. Mapping single molecule sequencing reads using basic local alignment with successive refinement (BLASR): application and theory. *BMC Bioinformatics* **13**: 238.

Chin CS, Alexander DH, Marks P, Klammer AA, Drake J, Heiner C, Clum A, Copeland A, Huddleston J, Eichler EE et al. 2013. Nonhybrid, finished microbial genome assemblies from long-read SMRT sequencing data. *Nature methods* **10**: 563-569.

Goodwin S, Gurtowski J, Ethe-Sayers S, Deshpande P, Schatz MC, McCombie WR. 2015. Oxford Nanopore sequencing, hybrid error correction, and de novo assembly of a eukaryotic genome. *Genome Res* **25**: 1750-1756.

Kurtz S, Phillippy A, Delcher AL, Smoot M, Shumway M, Antonescu C, Salzberg SL. 2004. Versatile and open software for comparing large genomes. *Genome biology* **5**: R12-R12.

Lee H, Gurtowski J, Yoo S, Marcus S, McCombie WR, Schatz M. 2014. Error correction and assembly complexity of single molecule sequencing reads. *biorxiv* doi:10.1101/006395.

Li H. 2013. Aligning sequence reads, clone sequences and assembly contigs with BWA-MEM. *arXiv preprint arXiv:13033997*.

Loman NJ, Quick J, Simpson JT. 2015. A complete bacterial genome assembled de novo using only nanopore sequencing data. *Nature methods* **12**: 733-735.

Phillippy AM, Schatz MC, Pop M. 2008. Genome assembly forensics: finding the elusive mis-assembly. *Genome biology* **9**: R55-R55.

Walker BJ, Abeel T, Shea T, Priest M, Abouelliel A, Sakthikumar S, Cuomo CA, Zeng Q, Wortman J, Young SK et al. 2014. Pilon: an integrated tool for comprehensive microbial variant detection and genome assembly improvement. *PLoS One* **9**: e112963.
