# Supplementary figures and images for: Canu: scalable and accurate long-read assembly via adaptive k-mer weighting and repeat separation
Source: Genome Res. 2017 May;27(5):722–36. doi: 10.1101/gr.215087.116 (PMC5411767; doi:10.1101/gr.215087.116)

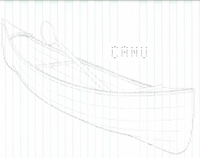

Supplement: Supplemental Material [file supp_gr.215087.116_Supplemental_Code.tar.gz › canu-1.3/logo.jpg]
